# Supplementary material for: Neural network and kinetic modelling of human genome replication reveal replication origin locations and strengths
Source: PLoS Comput Biol. 2023 May 30;19(5):e1011138. doi: 10.1371/journal.pcbi.1011138 (PMC10256156; doi:10.1371/journal.pcbi.1011138)
Supplement: S1 Text — Table A. Pearson correlation coefficients between experimental MRT and RFD profiles and their simulated MRTn and RFDn estimates obtained for the series of iteratively optimised IPLS In using RFD derivative for initialisation of I0. Results are shown for K562, GM06990, Hela and Raji cell lines as well as S. cerevisiae. At the 5th iteration none of the PCC increased (not shown). Fig A. Comparison between 〈RFD〉xx+l (blue) and vlΔlMRT(x) (red) (Eq (2)) assuming TS = 12h at different scale l. From (top) to (bottom), l = 100 kb, 200 kb, 500 kb and 1000 kb and fork speed values v are taken from Fig 2B. The same 20 Mb region of chromosome 1 as in Fig 2A is shown. Fig B. Comparison of K562 IPLSs optimised either from DNase I HS peaks (orange) or from the experimental ΔRFD peaks (blue). The obtained IM profiles are almost identical (PCC = 0.94). Fig C. Zoom on chromosome 1 for K562. Top: experimental (black) and simulated (red) MRT. Middle Top, experimental black) and simulated (red) RFD. Middle Bottom, input IM profiles. Bottom, Probability of activation. Fig D. Effect of single parameter variation on measurable targets in GM06990. Effect of the density of firing factors ρF (A,B,C); the average distance between potential origins dPO (D,E,F); the percent of random initiation r (G,H,I); the fork speed v (J,K,L), on the Pearson Correlation Coefficient (PCC) between simulated and experimental MRT (A,D,G,J) and RFD (B,E,H,K) profiles, and on T95 (red), T99 (orange) and T100 (green), the median times required to replicate 99% and 100% of the genome (C,F,I,L). Fig E. Effect of single parameter variation on measurable targets in Hela. Effect of the density of firing factors ρF (A,B,C); the average distance between potential origins dPO (D,E,F); the percent of random initiation r (G,H,I); the fork speed v (J,K,L), on the Pearson Correlation Coefficient (PCC) between simulated and experimental MRT (A,D,G,J) and RFD (B,E,H,K) profiles, and on n T95 (red), T99 (orange) and T100 (green), [file pcbi.1011138.s001.pdf]

# Supporting information

## Nomenclature

$[F_{free}]$ Average number of free firing factors (unitless)

$\rho_F$ Density of firing factors ($\text{Mb}^{-1}$)

$A_x(t)$ Probability for bin $x$ to have been activated at time $t$

$d_{PO}$ Mean distance between potential origins (kb)

$F_{free}(t)$ Time-dependent number of free firing factors

$I_0$ Starting IPLS profile of the iterative procedure

$I_1 \cdots I_4$ Intermediary IPLS profiles

$I_M$ Optimum IPLS profile

$I_M(x)$ Optimum IPLS profile at bin $x$

$k_{on}$ Bimolecular reaction rate between firing factors and potential origins ($\text{min}^{-1}$)

$L$ Total length of the considered genome (kb)

$MRT$ Mean Replication Time normalised between 0 and 1,(unitless)

$MRT_t(x)$ Mean Replication Time in time unit at bin $x$ (min)

$n(x)$ Number of potential origins at bin $x$

$n_e^a$ Density of potential origins estimated from MRT, RFD using a constant number of free firing factors (Eq. (8))

$n_e$ Density of potential origins estimated from MRT, RFD using the time-dependent number of free firing factors (Eq. (6))

$n_e^{exp}$ Density of potential origins estimated from MRT, RFD using the exponential dependency on MRT (Eq. (9))

$OE(x)$ Observed efficiency over the length of the S-Phase (unitless $\in [0, 1]$)

$r$ Random activation rate expressed as a percent of the total IPLS

$RFD$ Replication Fork Directionnality (unitless)

$T_S$ S-phase duration (min)

$v$ Fork speed ($\text{kb.min}^{-1}$)

| IPLS | K562 MRT | K562 RFD | GM MRT | GM RFD | Hela MRT | Hela RFD | Raji MRT | Raji RFD | *S. cer.* MRT | *S. cer.* RFD |
|---|---|---|---|---|---|---|---|---|---|---|
| $I_0$ | 0.81 | 0.79 | 0.74 | 0.77 | 0.78 | 0.74 | 0.70 | 0.77 | 0.60 | 0.71 |
| $I_1$ | 0.93 | 0.89 | 0.95 | 0.88 | 0.95 | 0.82 | 0.92 | 0.89 | 0.80 | 0.83 |
| $I_2$ | 0.98 | 0.91 | 0.98 | 0.91 | 0.97 | 0.85 | 0.93 | 0.90 | 0.87 | 0.84 |
| $I_3$ | 0.98 | 0.92 | 0.99 | 0.91 | 0.98 | 0.85 | 0.94 | 0.90 | 0.94 | 0.90 |
| $I_4$ | 0.98 | 0.92 | 0.99 | 0.91 | 0.99 | 0.84 | 0.93 | 0.88 | 0.96 | 0.91 |

**Table A.** Pearson correlation coefficients between experimental MRT and RFD profiles and their simulated $MRT_n$ and $RFD_n$ estimates obtained for the series of iteratively optimised IPLS $I_n$ using RFD derivative for initialisation of $I_0$. Results are shown for K562, GM06990, Hela and Raji cell lines as well as *S. cerevisiae.* At the $5^{\text{th}}$ iteration none of the PCC increased (not shown).

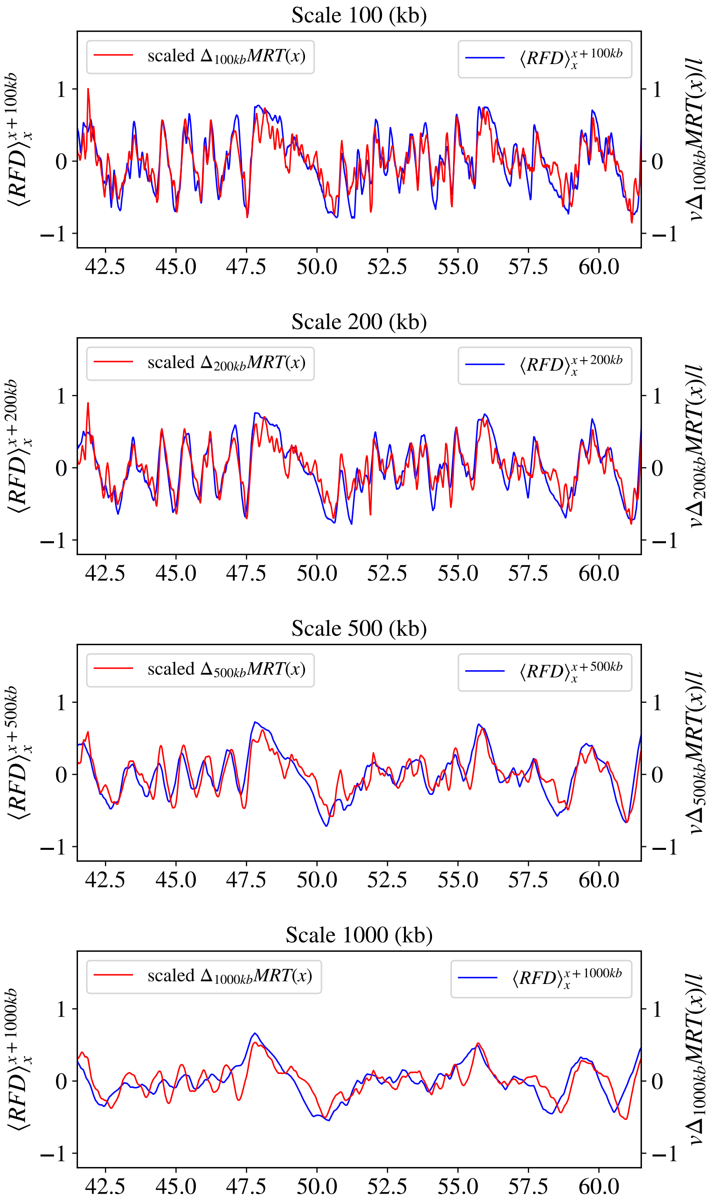


**Fig A.** Comparison between $\langle RFD\rangle_x^{x+l}$ (blue) and $\frac{v}{l}\Delta_l MRT(x)$ (red) (Eq. (2)) assuming $T_S = 12$h at different scale $l$. From (top) to (bottom), $l = 100$ kb, 200 kb, 500 kb and 1000 kb and fork speed values $v$ are taken from Fig 2B. The same 20 Mb region of chromosome 1 as in Fig 2A is shown.

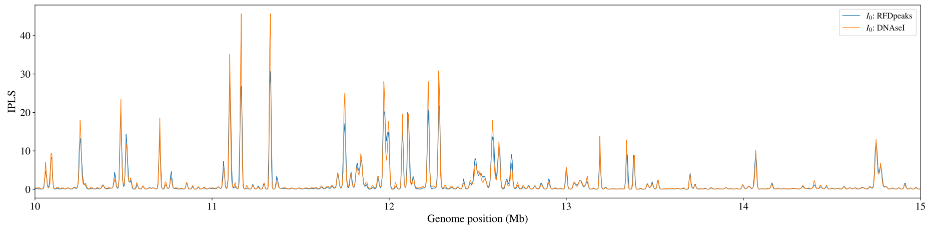


**Fig B.** Comparison of K562 IPLSs optimised either from DNase I HS peaks (orange) or from the experimental $\Delta RFD$ peaks (blue). The obtained $I_M$ profiles are almost identical (PCC = 0.94).

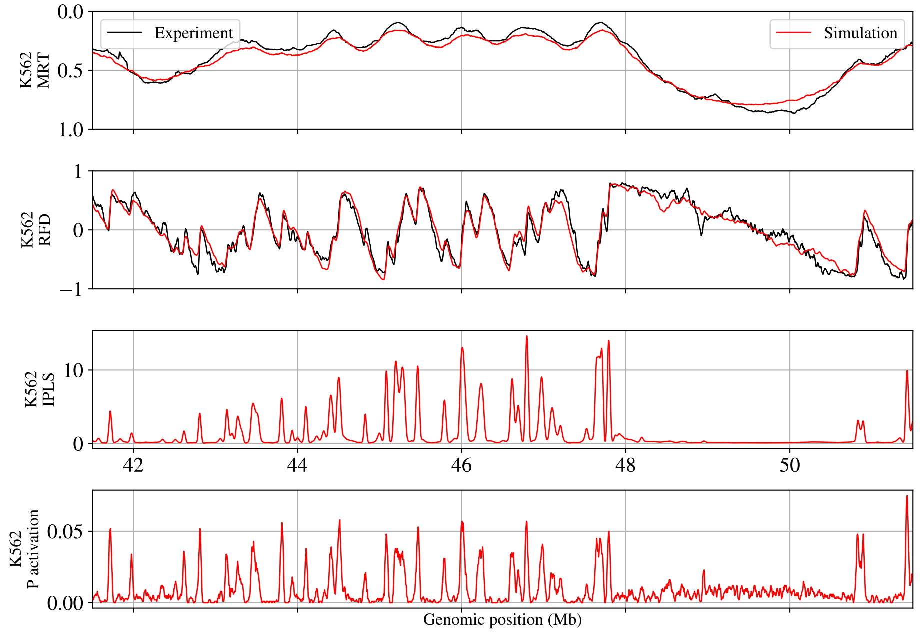


**Fig C.** Zoom on chromosome 1 for K562. Top: experimental (black) and simulated (red) MRT. Middle Top, experimental black) and simulated (red) RFD. Middle Bottom, input $I_M$ profiles. Bottom, Probability of activation.

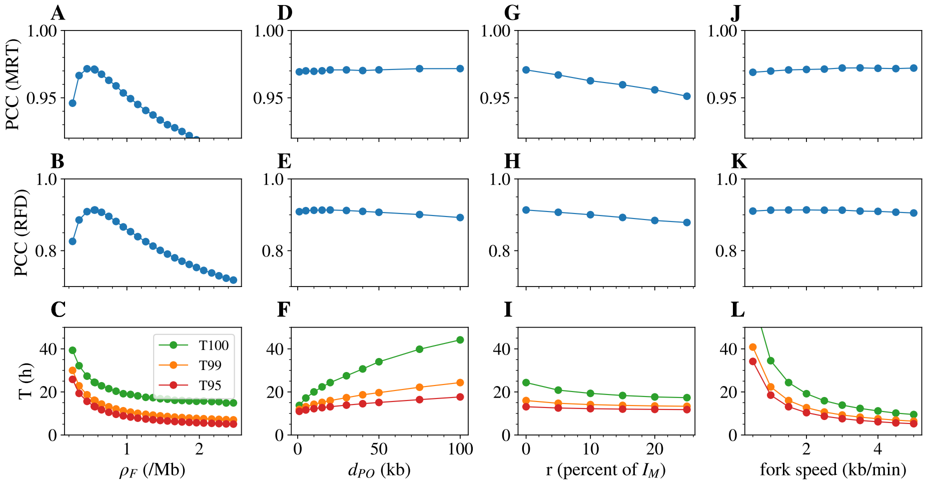


**Fig D.** Effect of single parameter variation on measurable targets in GM06990. Effect of the density of firing factors $\rho_F$ (A,B,C); the average distance between potential origins $d_{PO}$ (D,E,F) ; the percent of random initiation $r$ (G,H,I); the fork speed $v$ (J,K,L), on the Pearson Correlation Coefficient (PCC) between simulated and experimental MRT (A,D,G,J) and RFD (B,E,H,K) profiles, and on T95 (red), T99 (orange) and T100 (green), the median times required to replicate 99% and 100% of the genome (C,F,I,L).

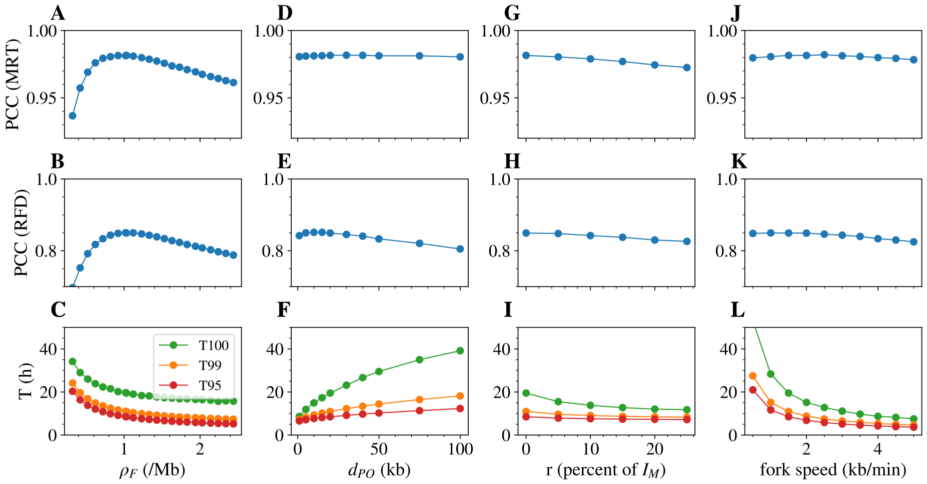


**Fig E.** Effect of single parameter variation on measurable targets in Hela. Effect of the density of firing factors $\rho_F$ (A,B,C); the average distance between potential origins $d_{PO}$ (D,E,F) ; the percent of random initiation $r$ (G,H,I); the fork speed $v$ (J,K,L), on the Pearson Correlation Coefficient (PCC) between simulated and experimental MRT (A,D,G,J) and RFD (B,E,H,K) profiles, and on n T95 (red), T99 (orange) and T100 (green), the median times required to replicate 99% and 100% of the genome (C,F,I,L).

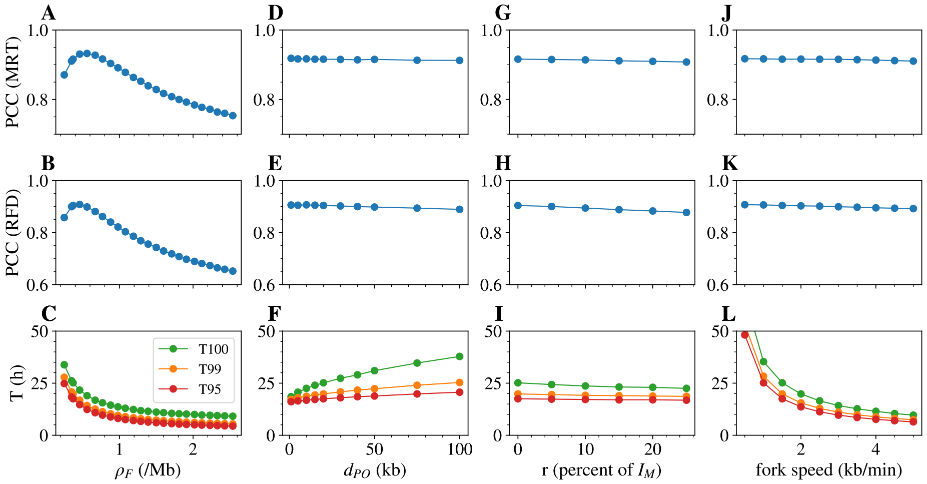


**Fig F.** Effect of single parameter variation on measurable targets in Raji. Effect of the density of firing factors $\rho_F$ (A,B,C); the average distance between potential origins $d_{PO}$ (D,E,F) ; the percent of random initiation $r$ (G,H,I); the fork speed $v$ (J,K,L), on the Pearson Correlation Coefficient (PCC) between simulated and experimental log2 E/L (A,D,G,J) and RFD (B,E,H,K) profiles, and on n T95 (red), T99 (orange) and T100 (green), the median times required to replicate 99% and 100% of the genome (C,F,I,L).

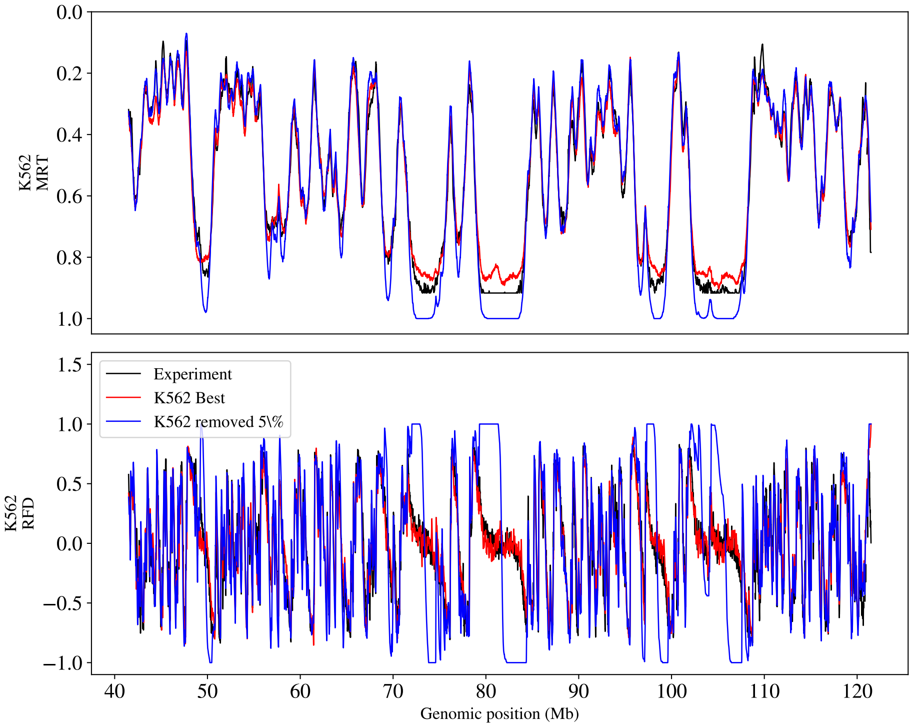


**Fig G.** (Top) Comparison of experimental MRT (black) with simulated MRT using the optimised IPLS $I_M$ (red) and after setting to zero the bins with the lowest $I_M(x)$ values corresponding to 5% of the total origin firing events (≈ 53% of the bins) (blue). (Bottom) Same comparison for the RFD profiles.

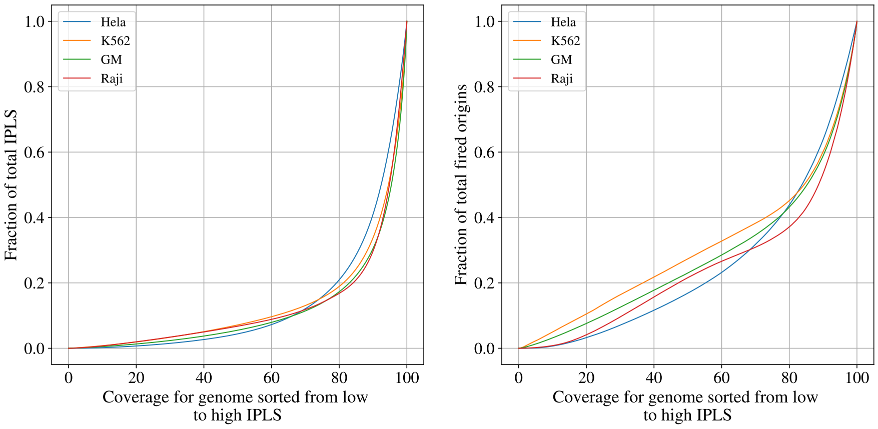


**Fig H.** Left: fraction of the total IPLS as a function of the coverage for genome sorted from low to high initiation. Right: fraction of the total probability of activation as a function of the coverage for genome sorted from low to high IPLS.

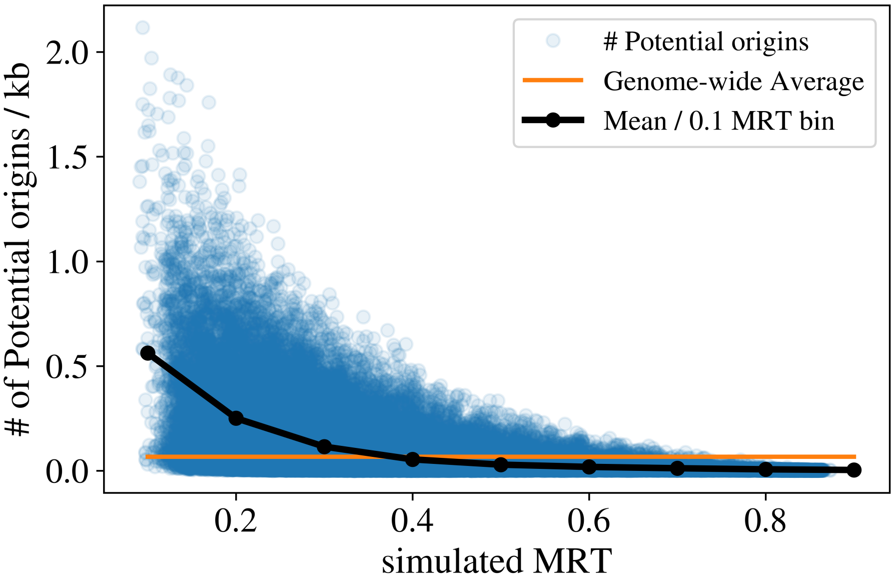


**Fig I.** Predicted number of potential origins per kb, computed for each 5 kb bin, vs. MRT in K562 replication simulations.

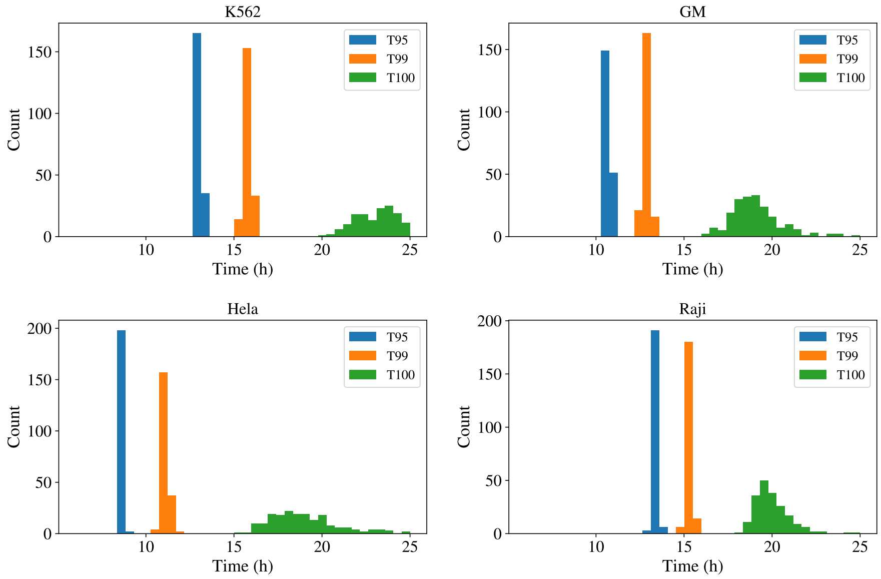


**Fig J.** Distribution of T95 (blue),T99 (orange) and T100 (green) replication times as defined in the text for the four different cell lines, as indicated.

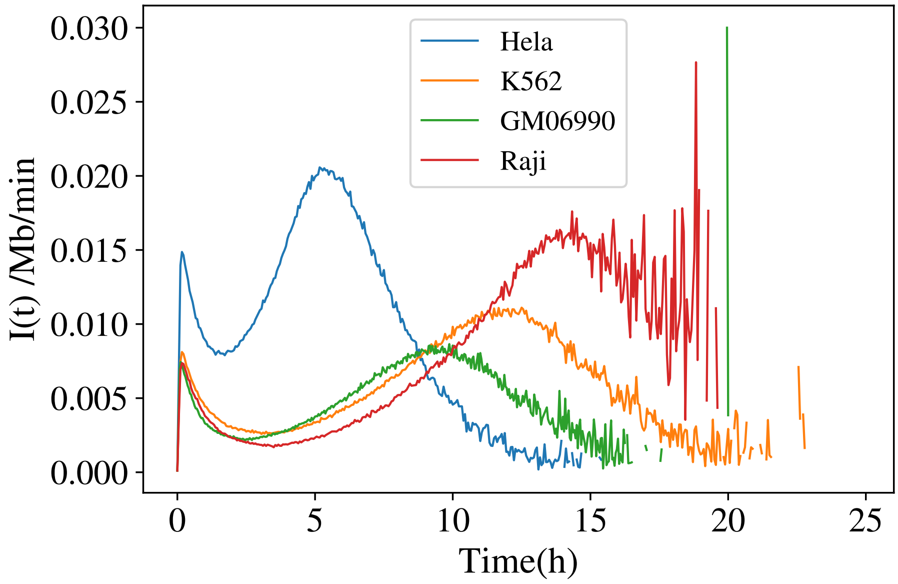


**Fig K.** Probability of initiation per length of unreplicated DNA per minute for the four indicated cell lines.

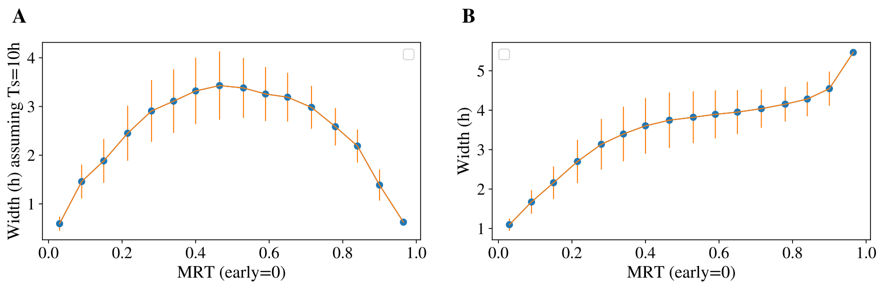


**Fig L.** Replication time (RT) variability as a function of MRT in simulated K562 replication. Blue dots and orange bars indicate the genome-wide average and range of values, respectively of RT variability. A) RT variability computed using the replicated genome fraction as a proxy for RT, plotted against MRT. B) RT variability using the true simulated time, plotted as a function of MRT.

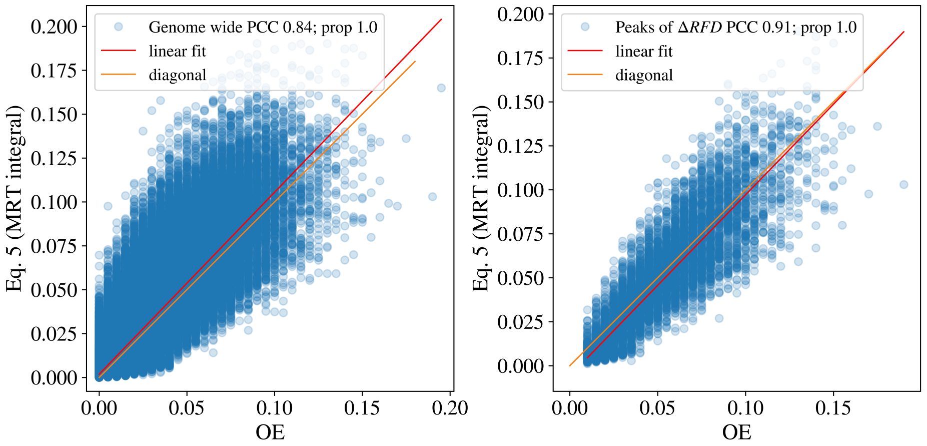


**Fig M.** Comparison of observed origin efficiency in K562 replication simulation, directly counted as the fraction of simulations in which replication started in a bin (OE), or computed as the right-hand side term of Eq. (5), genome wide (left) or restricted to the peaks of $\Delta RFD$ (right). Red line represents the linear fit and the orange line the first diagonal.

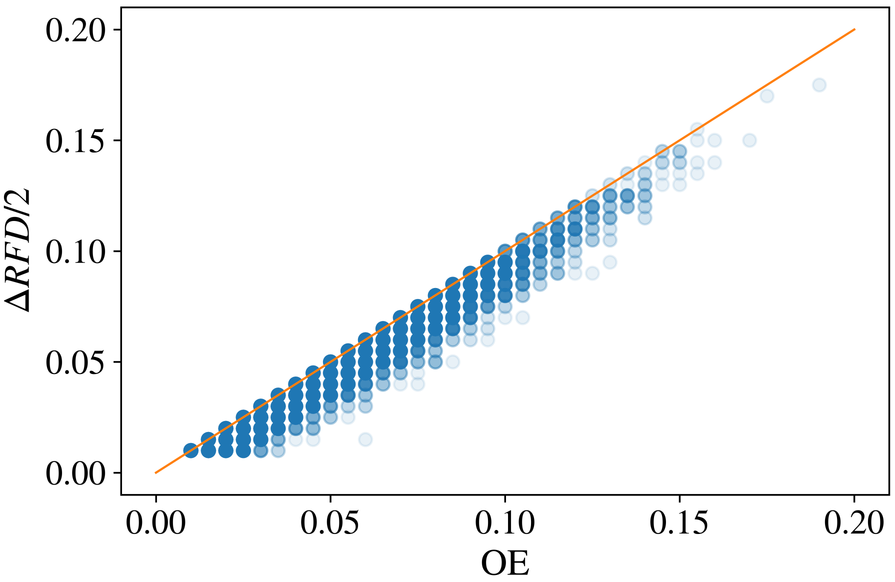


**Fig N.** Comparison of OE, directly counted as the fraction of simulations in which replication started in a bin (OE), with its estimation by $\Delta RFD/2$ restricted to peaks of $\Delta RFD$. The orange line is the first diagonal.

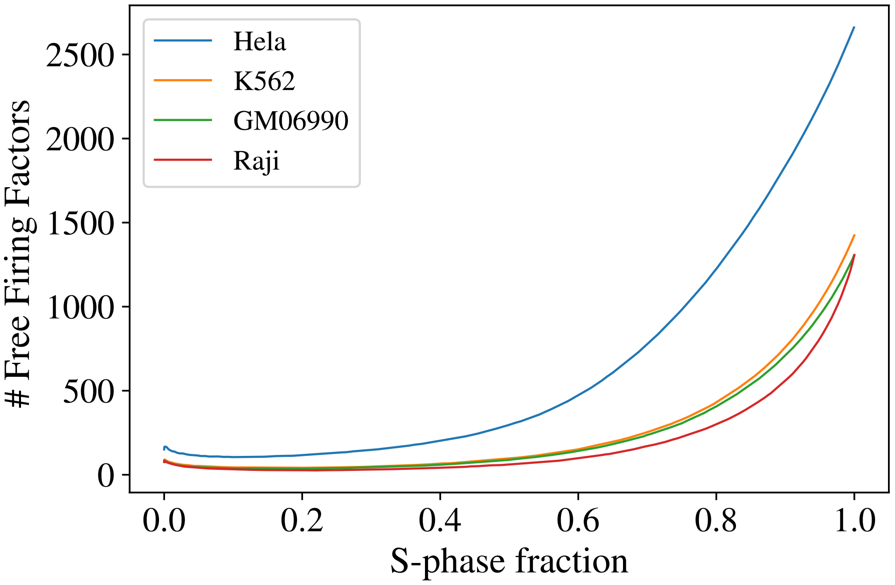


**Fig O.** Number of Free firing factors as a function of the S-phase fraction for the four indicated cell lines.

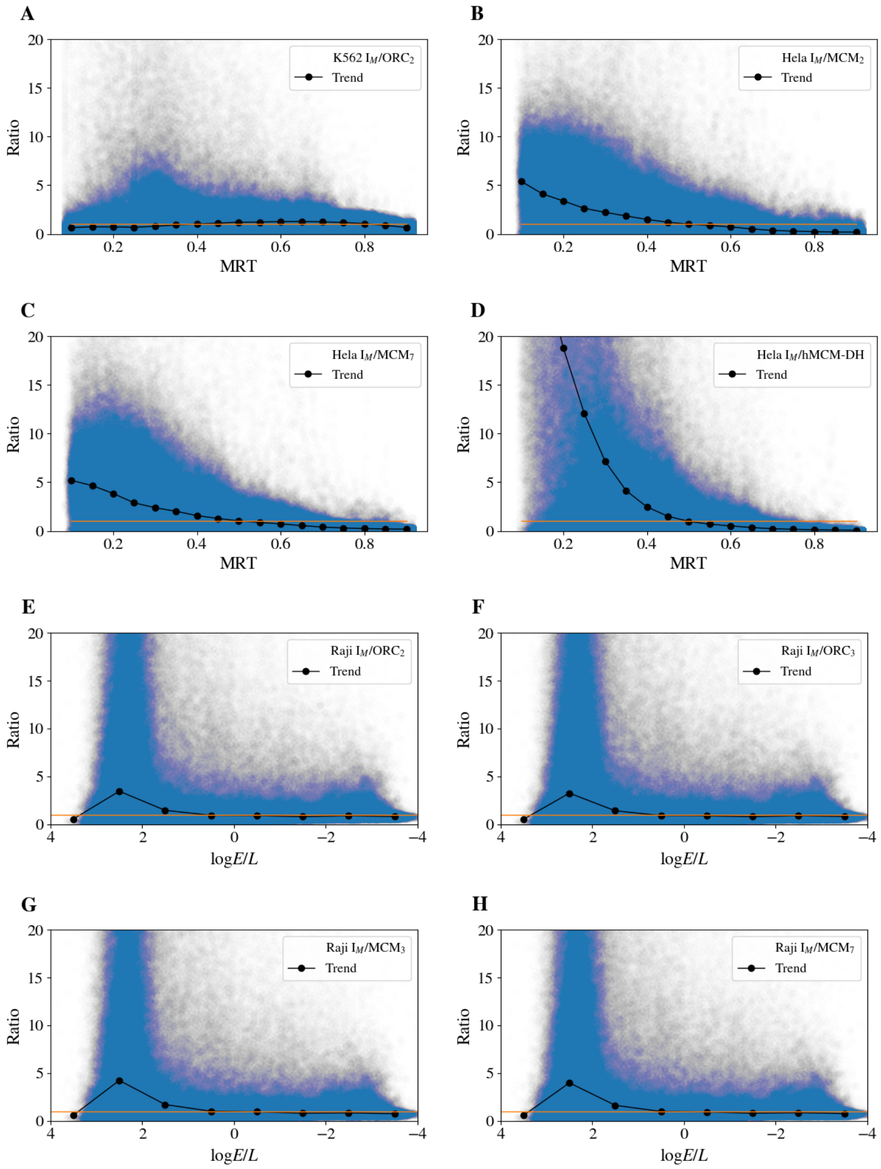


**Fig P.** A) $I_M(x)/ORC_2(x)$ ratio in K562 . B) $I_M(x)/MCM_2(x)$ ratio in Hela. C)$I_M(x)/MCM_7(x)$ ratio in Hela. D)$I_M(x)/hMCM\text{-}DH(x)$ ratio in Hela. E) $I_M(x)/ORC_2(x)$ ratio in Raji. F) $I_M(x)/ORC_3(x)$ ratio in Raji G) $I_M/MCM_3$ ratio in Raji H) $I_M(x)/MCM_7(x)$ ratio in Raji. Due to noise in MCM or ORC data all signals were smoothed with a 50 kb sliding window and normalized so that the median value over all the genome was one (orange line). For A-D, the black dotted lines indicate the median value of the ratios by 0.05 MRT steps. For E-H, the black dotted lines indicate the median value of the ratios by 1 $\log E/L$ steps.

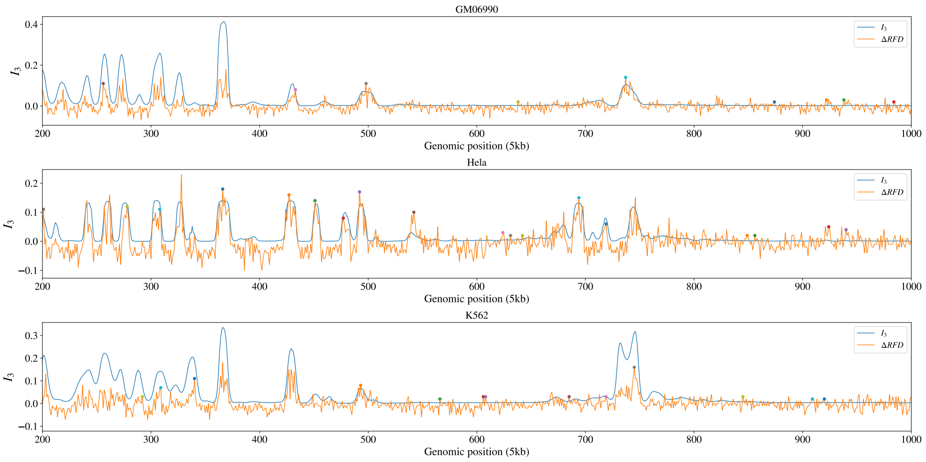


**Fig Q.** Peak detection of $\Delta RFD$ (dots on the top of the orange signal) overlayed with $I_M$ profile (blue curves) for the three indicated cell lines.
