# Supplementary material for: Neural network and kinetic modelling of human genome replication reveal replication origin locations and strengths
Source: PLoS Comput Biol. 2023 May 30;19(5):e1011138. doi: 10.1371/journal.pcbi.1011138 (PMC10256156; doi:10.1371/journal.pcbi.1011138)
Supplement: Attachment — Submitted filename: Reply to Email from elife boardFINAL.pdf [file pcbi.1011138.s002.pdf]

Reply to Email from elife board

Dear Dr Arbona,

Thank you for submitting the paper "Neural network and kinetic modelling of human genome replication reveal replication origin locations and strengths" for consideration by eLife. Your article has been reviewed by 3 peer reviewers, and the evaluation has been overseen by a Reviewing Editor and a Senior Editor.

Comments to the Authors:

We are sorry to say that, after consultation with the reviewers, we have decided that this work will not be considered further for publication by eLife.

Although the reviewers appreciated the usefulness of the approach and the quality of the work, the consensus of the panel is that it is more appropriate for a more specialized audience than that of eLife.

Our response :

We are happy that the board appreciated the usefulness and quality of the work. We are conscious that modelling DNA replication is a specialized field and may not be immediately appealing to a broad audience, but we remain convinced that such approaches are required to correctly interpret experimental datasets on DNA replication and epigenetics and therefore are of fundamental interest to a broad audience.

The work presents progress on two fronts: the computation inference of replication initiation kinetics from experimental replication kinetics data and the biological inference of the mechanisms that regulate replication initiation efficiency. The computational approach is an advance over previous, more brute-force, approaches, but it does not achieve markedly better results and thus seems to be more a technical advance than a conceptual one.

Our response :

It is not entirely clear to us to what "previous, more brute-force approaches" the board is referring to.

1. Did the board refer to <u>previous approaches to invert experimental data</u> into the IPLS?
As already explained in the discussion, the previous approaches by De Moura et al (2010), Baker and Bechhoefer (2014), and Bazarova et al (2019) either require a non trivial optimisation that may be feasible with the yeast but not the human genome, given its size, or a Bayesian analysis that so far was limited to sets of 3 origins in yeast. Therefore, our inversion approach, which infers an IPLS that predicts the MRT and RFD profiles <u>of the entire human genome</u>, is clearly far better than the three previous approaches.

2. Did the board instead refer to <u>previous approaches to predict experimental replication profiles</u>?
Gindin et al (2014) used a similar kinetic model of replication to test different epigenetic features, such as DNAse I HSS, transcription factor binding sites, or histone modifications, as estimates of the initiation probability landscape (IPLS). DNase I HSS profiles produced the best match with experimental MRT profiles (Pearson correlation between simulated and experimental MRT of 0.865). In our study,we inverted the experimental data to objectively <u>infer</u> an IPLS that produced a PCC of 0.98, which is close to perfection, and certainly much better than 0.865, <u>without making any assumption</u> on the epigenetic features that defined this IPLS. Furthermore, the Gindin paper did not make any prediction about experimental RFD profiles, which were not available at the time, and are of much higher resolution that experimental MRT profiles. Using DNAse I HSS, we found a PCC of only 0.70 between simulated and experimental RFD profiles; using our inferred IPLS, the PCC raised to 0.92, which in our opinion is an <u>even more dramatic improvement</u> over this previous approach.

To further substantiate that our predictions are substantially better than in Gindin et al, we have now analyzed which fraction of the predicted potential origins and actual initiation events, using either the DNAse I HSS profile or our optimized IPLS, fell within the initiation zones reported in Petryk et al.
In HeLa cells, these initiation zones cover 11% of the genome. Using DNAse I HSS, 35% of the potential origins and 23% of initiation events fell within these zones. Using our IPLS, these numbers increased to 46% and 35%, repectively. In GM06990, in which the initiation zones cover 7% of the genome, these numbers were 23% and 16% using DNAse I HSS but increased to 43% and 30% using our IPLS.
As an even more stringent test of the predictive capacity of our IPLS compared to DNAse I HSS, we selected the strongest 2% experimental and simulated RFD upshifts and measured their overlap. Using DNAse I HSS, the overlap was 11% for HeLa, 11% for GM06990 and 18% in K562. Using our IPLS, these overlaps increased to 43%, 51%, and 60%, respectively, a dramatic 3-5 fold increase. These results clearly demonstrate that our approach does produce markedly better results than the previous DNAse I HSS approach. These new results are presented lines 313-323 of the revised manuscript.

In summary, we vigorously contest the argument that our work " does not achieve markedly better results" *than previous approaches.*

The biological insight-that origin usage is influenced by a combination of origin licensing efficiency, origin firing efficiency and origin passivation-is well-supported by the authors analysis, but is consistent with much previous work and, again, cannot be seen as a conceptual advance. The work offers the promise of more specific biological insight, such as indication of which chromatin contexts contribute to regulation of origin licensing efficiency and origin firing efficiency. However, it is unclear if the currently available ORC and MCM mapping data, upon which such analyses would depend, is of adequate resolution or quality to draw meaningful conclusions.

Our response :

What determines origin usage is certainly a complex matter. There is not yet a clear consensus on the respective contributions of origin licensing, firing and passivation.

Yang, Rhind and Bechhoefer (2010), based on a time-course analysis of S. cerevisiae genome replication, proposed "a model where an origin is composed of multiple initiators, each of which fires independently and identically" *and suggested that* "a good candidate for the initiator is the minichromosome maintenance (MCM) complex"*. In a follow-up study, Das et al (2015) reported additional evidence in S. cerevisiae for a multiple-MCM model positing that* "MCM complexes are stochastically activated with similar probabilities across the genome"*. On the other hand, they acknowledged that* "The correlation we see between MCM ChIP-seq signal and replication timing is <0.5, consistent with MCM number having a significant but not exclusive role in regulating origin timing." *More recently, Foss et al (2021) mapped MCM binding sites in budding yeast, fission yeast, mouse and human HeLa cells using Mcm-ChEC-seq. They concluded that* "with a few notable exceptions, the pattern of replication timing in S phase in all four species is largely predictable simply on the basis of the pattern of helicase loading in the preceding G1."

In contrast to this model, Sugimoto et al (2018) mapped the distribution of MCM7 by ChIP-seq in HeLa cells and, comparing the data with SNS-seq, concluded that "a large number of MCMs are uniformly loaded in a genome-wide manner, irrespective of chromatin status, but only a small proportion are passively fired in chromatin regions with an accessible, open structure"*. Their results therefore argue against a model whereby MCM abundance determines the probability of initiation.*

Very recently, Li et al (2023) reported (after the submission of our original manuscript) a near-nucleotide resolution landscape of MCM DH footprints that revealed that MCM DH abundance *increases* *from early to late replication domains. In early replicating domains, MCM DHs form clusters of mean size 30 kb that align very well with OK-seq and ORM initiation zones. In later-replicating domains, MCM DHs form much larger (up to several Mb) and denser clusters. IZs. The authors conclude that their data* "support the notion that neither the size nor the signal intensity of the MCM-DH clusters correlates with origin firing efficiency, arguing against the hypothesis that efficiency is determined by the number of the MCM-DHs loaded at a particular site or IZ."

We have now analyzed the Li et al data and consistently found that they are *anticorrelated* *with MRT and RFD data. We quote this study lines 125-176 in the Introduction and present our new results and the comparison with alternate MCM datasets lines 558-622, Table 1, Table 2 and Fig. S16. These results are further discussed lines 644-655, 718-720, 733-735, 746-748.*

While we have no definitive argument to evaluate the respective quality of the three HeLa cell datasets, the excellent alignment of the early MCM DH clusters of Li et al with OK-seq and ORM initiation zones is impressive. We stress the disparities of the three datasets in the manuscript and we conclude that the inability of any of these MCM genomic profiles (including the data of Foss et al; our results contradict the conclusions of these authors) to correctly predict MRT and RFD genome-wide implies that the strict origin density model must be abandoned in favor of a variable origin affinity model.

Several studies in yeast (discussed lines 721-728) have suggested that orthogonal mechanisms to MCM loading (recruitment of DDK by Ctf19 or Fkh1/2, modulation of Fkh1-origin binding by Rpd3 and counteraction of DDK-mediated phosphorylation by Rif1-PP1) regulate origin usage *after* *the origin licensing step. Of note, recent studies also imply Rif1-PP1 in replication timing regulation in higher eukaryotes, which might explain the delayed firing of dense MCM DH clusters observed by Li et al (further discussed lines 729-735).*

In summary, despite clear evidence that origin usage is influenced by a combination of origin licensing efficiency, origin firing efficiency and origin passivation, previous studies comparing genome-wide replication time and MCM binding profiles of (Yang, Das, Foss) have put emphasis on MCM loading (i.e .origin licensing) as the primary mechanism of replication timing regulation. In contrast, our results and the novel MCM DH mapping study of Li et al clearly show that the firing probability of MCM complexes is *not uniform* *in time and space.*

We acknowledge that our conclusions might be limited by the quality of MCM mapping data but the same could be said of all previous studies that related replication timing to MCM density. On the other hand, our analysis of MRT and RFD profiles, which we demonstrate are highly consistent with each other, has allowed us to infer an IPLS of much better quality than previously modelled by DNAse HSS or other epigenetic features. Please note that *ORC data are better correlated to our IPLS than MCM data*. *An ORC-based IPLS predicts MRT and RFD reasonably well (PCC of 0.87 and 0.75, respectively), in contrast to MCM data (Table 1). Nevertheless, our IPLS still achieves better PCCs of 0.98 and 0.92, respectively. Whether this only reflects imprecision of ORC data or additional contributions of post-licensing mechanisms may of course be debated. However, this uncertainty does not prevent us to investigate the epigenetic determinants of our IPLS, whether they operate at licensing, firing, or both steps. This will be the subject of a future report.*

Reviewer 1 (Public Review):
Arbona et al. present a mathematical approach to derive the genome-wide distribution of DNA replication initiation events from two different types for replication kinetics data: mean replication time (MRT, aka T(rep)) and replication fork density/directionality (RFD). Formally, MRT and RFD are mathematically equivalent, but the authors show that, due to different sources

of noise in the respective datasets, the two data type contain complimentary information. The approach will be a use tool for extracting firing probabilities from replication timing data. However, there are several issues that could be addressed to improve the manuscript.
The first issue is that, although the authors claim to ”infer an origin licensing landscape”, they are actually inferring the probability of origin firing, not licensing. Their potential origin density landscape (PODLS) is equivalent to the previously described initiation probability landscape (IPLS). They are not obliged to use the same terminology (although it would help consolidate the concept), but they do need to acknowledge that the parameter that they infer from their model is initiation probability, not licensing probability. The fact that they set the probability of initiation of licensed origins to a genome-wide constant in their initial model-thus setting licensing probability equal to initiation probability-is immaterial; the event that their model captures is initiation, not licensing.

Our response :

We agree that the PODLS we infer seems equivalent to the IPLS as defined by Gindin et al. and for simplicity we have abandoned the PODLS acronym and now use IPLS instead.

However, there is one important difference between our modelling framework and the one of Gindin. While Gindin et al directly use their IPLS as a continuous initiation probability landscape, at the single cell level potential origins are discrete objects (MCM DHs) that occupy discrete positions on the genome. We previously demonstrated that explicit discretization of the potential origins in the modelling framework has important consequences on some kinetic parameters of DNA replication (Arbona et al 2018). We therefore incorporated this discretization of potential origins in the current work. The way we do this is to draw, for each realisation of the simulation, a fixed number of discrete elements from a continuous, arbitrary IPLS and we assign to each object the same intrinsic probability of firing, Therefore, in our model, heterogeneity in local firing probability only arises from heterogeneity in potential origin density.

We understand that the use of a new acronym may be confusing and we now use IPLS through the manuscript. We simply state in the Discussion, lines 641-644, that " In our modelling framework, the IPLS is equivalent to a potential origin density landscape where all potential origins have the same elementary probability of firing, independently of genomic location and time."
We hope this will clarify the above point.

The second issue is that the biological implications of the work seems incomplete and incremental.
The authors compare their inferred initiation probabilities with origin licensing data (MCM distribution data) and come to the conclusion that the probability of initiation of licensed origins is not a genome-wide constant, implying that some aspect of chromosome metabolism regulates the probability of initiation of licensed origins. While this concussion is compelling, it cannot be said to be unexpected; heterochromatin has been long known to inhibit origin initiation. Given the rich resources available that characterize chromatin structure in the cell lines analyzed in this study, the obvious and straightforward next step would be to correlate their observed heterogeneity of origin initiation with chromatin structure. As it is, the manuscript offers a useful technical advance, but little in the way of novel biological insight. A compounding factor is that the quality of the available MCM distribution data is questionable, meaning that even if the suggested analysis was done, it might well not support robust novel conclusions about the role of chromatin structure in regulating origin initiation.

Our response :

Please see our detailed response above. Despite clear evidence that origin usage is influenced by a combination of origin licensing efficiency, origin firing efficiency and origin passivation, previous

studies comparing genome-wide replication time and MCM binding profiles of (Yang, Das, Foss) put emphasis on MCM loading (i.e. origin licensing) as the primary mechanism of replication timing regulation. In contrast, our results clearly show that the firing probability of MCM complexes, whose distribution was mapped in previous studies including Foss et al, is <u>not uniform</u> in time and space.

We acknowledge that our conclusions might be limited by the quality of MCM mapping data but the same could be said of all previous studies that related replication timing to MCM density. Furthermore, the analysis of the new data of Li et al (2023), published after our initial submission, further support our conclusions. Their finding that MCM DH density increases from early to late replicating domains seems furthermore significant given the impressive alignment of the short MCM DH clusters in early-replicating domains with early-firing OK-seq and ORM initiation zones, which gives us confidence that these data are of good quality.

Importantly, ORC data better predict MRT and RFD than MCM data (Table 1). Yet, our optimal IPLS achieves even better predictions (PCCs of 0.98 and 0.92, respectively). Whether this only reflects imprecision of ORC data or additional contributions of post-licensing mechanisms that favor activation of ORC-proximal MCM DHs may of course be debated. However, this uncertainty does not prevent us to investigate the epigenetic determinants of our PODLS, whether they act at the licensing step, or firing step, or both. This will be the subject of a future report.

Reviewer 1 (Recommendations for the authors):

The authors note that published SNS- and Bubble-seq initiation datasets do not serve as useful PODLSs, which is consistent with the datasets being noisy and with the well known lack of concurrence between them. It would be useful to examine the more recent initiation data from Wang (2021) which was reported to work well when used as an IPLS for the Gindin model.

We have now used the Wang et al optical replication mapping (ORM) data, obtained in HeLa cells, as an IPLS in our simulation framework. We obtained a PCC between predicted and experimental HeLa MRT data of 0.87, very close to the PCC reported by Wang et al (0.85). However, the PCC was only 0.64 for the RFD data. To take into account that ORM data reflect observed rather than intrinsic origin efficiencies, we combined them with MRT data to infer an IPLS as we did with SNS- and Bubble-seq data. The obtained PCC between predicted and experimental MRT and RFD data raised to 0.92 and 0.71, respectively. Although these numbers were not as good as with our optimal IPLS (0.98 and 0.85, respectively), they were clearly better than those obtained with SNS- and Bubble-seq data. Our approach therefore underlines the satisfying, joint consistency of ORM with Repli-seq and OK-seq data. These new results are presented lines 549-556 and Table 1.
We also computed the direct correlation of ORM data, or other experimental datasets, with our optimal IPLS. The results are presented Table 2 and lines 572-584. The best correlations were obtained for ORM data.

Lines 66,7: Wang (2021) also showed direct evidence for dispersed initiation between efficient IZs.

We have added this reference (line 80 and line 84).

Line 89: Foss (2021) also suggested that the variable density of potential origins along the genome may determine MRT.

We have added this reference (line 103).

Line 350: The reduction in T(width) in late S seen in the Zhao paper is due to inappropriately normalized data. Specifically, they normalize all of their replication kinetics curves to go from 0% to 100%. Superficially, this normalization makes sense, because every cell is expected to have completed replication by the 16th fraction of the FACS distribution. However, in reality, there is significant noise in the sorting of the cells, especially in the late-S-G2-mitosis region of the FACS plot. So the assumption that all the cells in Fraction 16 have completed S is invalid. This noise is evident in the heat maps in Figure 1c of the Zhao paper. It shows measurable signal from late replicating regions in Fractions S1, S2 and even S3. These are cells that were labeled is late-S but divided before the FACS sort, ended up as G1 cells and spread out through S1, S2 and S3. The noise in the Takahashi paper comes from the fact that the position in S has to be inferred from the fraction of the genome replicated, which in turn is based on quite sparse single-cell sequence, but the effect is the same.
The problem with the normalization can be seen in first row of Figure 3b of the Zhao paper. Taking the mESC data, for instance, the red (early), blue (early mid), green (late mid) replication-kinetics curves all plateau at 100% replicated well before the end of S, and these curves get progressively less steep, indicating a broadening of their firing time distribution. However, the earliest black curves just barely make it to 100% by the end of S (arbitrarily set to 10 hours) and the later black curves should not reach 100% at all, but are forced to do so by the normalization. To get a feel for what the properly normalized data would look like, see Figure 3B in Yang (2010) https://www.ncbi.nlm.nih.gov/pubmed/20739926, which described a comparable analysis of yeast replication kinetics data.
The affect of the inappropriate normalization can also be seen in the second row of the same Zhao figure. Again looking at the mESC data, it is apparent that the red, blue, green and early black data show the expected increase in T(rep) and T(width), and that only the later black data, which is the data that is affected by the inappropriate 0-to-100% normalization, show a drastic (and artifactual) reduction in T(width).

Line 352: Likewise, the authors observed reduction of T(width) in late S may be an artifact of assuming constant S-phase length.

We agree with the referee's criticism of the Zhao et al data. However, experimental caveats do not apply to our simulations.

We have now found that the reduction of Twidth in late S phase was a consequence of using the replicated genome fraction as a proxy for the real time. We initially did so in order to process the data in exactly the same manner as the Repli-seq data in Zhao et al. However, our simulations also give access to the real simulation time. We have now compared the variations of Twidth, as measured either by the replicated genome fraction or by the true simulated time, along S phase progression. It is now clear that the true replication time dispersion increases until the end of S phase, as expected for stochastic origin firing. Therefore, our (and other investigators') previous results, that suggested a decrease in replication time variability in late S phase, were an artefact due to the false assumption that the replicated genome fraction can be used as a proxy for the real time.

These new results are presented lines 432-450 and on Fig S12.

Line 364: IE, as defined, is always 1, as stated on line 372. A more useful definition would be over a fixed t, and might be better termed firing probability.

We had defined IE line 364 as " the efficiency that would be observed, in the absence of passivation, over the entire length of S phase." This is never 1. IE = 1 only when an origin is given an infinite time to fire. Whatever the fixed (but finite) t chosen to define IE, there is always the possibility that the origin has not yet fired at time t. Therefore IE is never 1, unless t is infinite.

To avoid possible confusion, we have rewritten this paragraph (lines 452-460) as follows :

"Origin firing can be prevented by context-dependent passivation from nearby origins. It is therefore important to distinguish between the local density of potential origins within a bin, which in the origin density model is directly proportional to the IPLS, and the observed origin efficiency (OE), which is the fraction of chromosomal copies in the cell population in which an origin fired in that bin. OEs can be directly measured by SNS-seq, Bubble-Seq, ORM or indirectly by RFD upshifts. In the following we will derive a mathematical relationship that relates OE to IPLS and MRT. This allows us to predict the IPLS, the input of our simulations, directly from MRT and either SNS-seq, Bubble-Seq, ORM or OK-Seq experiments."

Line 461: Wittig (2021) does not report that firing factor abundance decreases during S phase. They report that TIRCC concentration decreases at the G1/S transition and then stays stable during S.

The title of this study is "The CRL4DTL E3 ligase induces degradation of the DNA replication initiation factor TICRR/TRESLIN specifically during S phase". Although Figure 1 shows indeed that most of the degradation occurs upon entry into S phase, experiments shown in Figure 2, as the authors conclude, "demonstrate the decrease in both insoluble and total TICRR levels is due to TICRR protein degradation via a cullin-dependent pathway during S phase". Experiments in Figures 5 and 8 also show that PCNA and CDC45 are required for this degradation. Therefore, even though we agree that most of the degradation occurs at the start of S phase, it is clear that degradation occurs in response to DNA synthesis, so that the earliest-firing origins experience a higher concentration of this limiting factor than origins that fire during the rest of S phase.

Reviewer 2 (Public Review):
This paper is an attempt to improve the analysis of DNA replication data in order to better infer the position and efficiency of replication-origin initiation. The analysis, which uses neural networks, improves the fit with data and concludes that origin efficiency is set at both the origin-licensing and origin-initiation steps. In other words, they argue for an ”origin-affinity” model of DNA replication where the likelihood to fire is controlled by factors beyond the density of either ORC or MCM complexes. The null hypothesis, that initiation is controlled primarily by ORC or MCM density, is termed here the ”origin-density” model. The paper is a generally skillful combination of simple modeling assumptions and numerical simulations to come up with useful formulas relating observed quantities (MRT(x) and RFD(x)) to quantities of interest [ne(x) = estimated number of MCM DHs in bin x]. The main conclusions that (i) kon and/or Ffree may be higher in early S phase while MCM DHs are near their steric limit (supporting a scenario ”in between” the origin-affinity and origin-density models) and (ii) MCM DHs that remain closer to ORC sites are more likely to fire than ORC-distal ones are interesting and should inspire follow-up work. For that reason and because it generally refines efforts to quantitatively model DNA replication, this paper should make a substantial contribution to the literature.
One worry is that Eq. (6) predicts an inverse dependence of ne(x) on MRT(x). But the simulations (Fig. 8b) imply a stronger, perhaps-exponential decay. The difference is tentatively ascribed (Line 419) to the variation in Ffree(t) (Fig. S11). Since this is being explored in simulations, it seems that one should be able to say something more certain here. Maybe one can analyze in more detail data from the time Ffree is more nearly constant to start to isolate this dependence?

To answer this question, we have modified our calculations in Eqns 4 to 6, so as to take into account the time-dependent variations of Ffree. We therefore replaced [Free] MRT(x) by Integral(Free(t),0,MRT(x)). We now observe that the data are much better captured by the novel Eq 6. (Figure 8 A, B).

In experimental practice, however, one wishes to infer an IPLS from observed origin efficiencies using MRT data without knowledge of the molecular nature and temporal variations of Ffree. Fortunately, we made the empirical observation that under the (false) assumption that Ffree is constant, the data are best captured by the exponential decay law (Figure 8 C). We have no explicit rationalization for this finding. Using the exponential rather than the inverse law must somehow compensates for neglecting the temporal variations of Ffree. The simulations verify that this empirical fit gives good inferences of intrinsic origin efficiencies.

The entire section has been rewritten accordingly (lines 452-526) and the results are shown on a new Figure 8.

Reviewer 2 (Recommendations for the authors):
1. There are a large number of symbols (some with several variants) that are defined in numerous places throughout the text and then re-used later. It would help the reader to have a table listing and briefly defining them all.

A nomenclature of all the symbols used in the text is now given in the Supplementay Information. A note has been added at the start of the Results section to indicate this.

2. The authors dismiss numerical differentiation, stating that "the derivative of a noisy pro-file is ill-defined and numerically unstable to compute." The statement is certainly true of naive numerical differentiation algorithms. But there are many sophisticated algorithms (differential quadrature, Savitzky-Golay, wavelets, ...) that can separate the low-frequency true low-frequency profile variations from high-frequency noise. Using such an algorithm could lead to a nicer presentation of the RFD data. Changes in analysis methods, however, probably wouldn't change the conclusion that MRT gives large-scale (50-100 kb) information on origin positions while RFD gives complementary small-scale (5 kb) information.

We have rewritten this section and tried to better justify our approach lines 176-180 as follows :

"Equation (1) can be checked using experimental data. However, the derivative of experimental signal is ill-defined due to noise; slopes cannot be estimated pointwise as they are numerically unstable. This can be circumvented by data smoothing at the expense of resolution. Here, we noted that Eq. (1) can be integrated at point x over a length l leading to: (Eqn 2)"

We have not attempted to use the sophisticated algorithms mentioned by the Referee as we agree that this change in analysis methods would not change our conclusions.

3a. The discussion of the role of random initiation events (the parameter r0) could be clearer. On Line 276, the authors state that the optimal value is r*=0%, which seems to imply that there is no initiation outside the initiation zones (IZs). But Line 303 states that r=5% was "necessary to fully account for experimental data". It would help for the authors to explain better and thus reconcile these statements. (Is it that r*=0 gives the best fit within IZs but r=5% gives the best fit outside IZs, without altering significantly the fit inside the IZs?)

We agree that this discussion was difficult to follow without a clearer explanation of the input and output of the neural network optimization procedure and we have entirely rewritten this section. The network is initiated with a given, starting I + r function that is optimized to produce a continuous IPLS that can no longer be decomposed simply into peaks of "localized" initiation superimposed on a constant background of "random" initiation.

We therefore decided to 1) delete some confusing sections of our earlier version and rewrite a simpler description of r parameter optimization (lines 359-369); 2) extensively characterize the*

resulting IPLS by computing the cumulated share of the IPLS and the cumulated share of actual initiation events, as a function of the genome coverage ranked by increasing IPLS values (lines 370-379, Figure S8).

These new results show that although 80% of the IPLS was concentrated within 15-20% of the genome, this genome fraction only gave rise to 60% of initiation events, the remaining 40% being nearly uniformly distributed over the rest of the genome. These dispersive initiation events were essential to fully account for MRT and RFD data, since setting to zero the lowest IPLS bin values totalling 5% of the IPLS (and covering 53% of the genome) resulted in a decreased correlation with RFD from PCC = 0.92 to PCC = 0.82, and failed to capture the RFD and the MRT of extended late replicating regions (Figure S7).

The significance of this prevalent dispersive initiation is further discussed lines 681-688.

3b. A related question: It would help if the authors could also relate the random-initiation parameter to other quantities. For example, r=5% is described as small. This is certainly small in the sense that the rate is just 5% of that observed typically in IZs. But IZs are only part of the genome. With r=5%, what fraction of origins in a cell cycle are activated within IZs and what fraction is activated by the ”random” initiation outside IZs?

We have now performed this analysis and found that in HeLa, 46% of potential origins and 35% of actual initiation events in our simulations are found within the IZs reported in Petryk et al, which cover 11% of the HeLa genome. These numbers are 43%, 30% and 7%, respectively, for GM06990. Therefore, about two-thirds of initiation events actually occur outside of predominant IZs, consistent with the initial suggestion by Petryk et al that a large fraction of initiation events must occur in a dispersive manner outside of predominant IZs, and the direct observation by single molecule analysis of such dispersive initiation outside "master" IZs (Blin, Wang).

This novel analysis is presented lines 318-328 and, along with the novel data reported lines 370-379 and Figure S7, further discussed lines 681-688.

4. I did not understand precisely the statement (Line 381) that the ”IM profile is normalised to1”. The authors should define the normalization more precisely.

We changed to : IM profile is normalised so that its sum is equal to 1 (line 480).

Reviewer 3 (Public Review):
Replication fork directionality (RFD), obtained by Okazaki fragment sequencing (OK-seq), and mean replication time (MRT), obtained by S-phase sequencing, are two complementary approaches to measure global genome replication kinetics. The authors present a simple mathematical relationship between MRT and RFD, and they show that, while highly congruent, the two types of data provide optimal insights into replication at different spatial resolutions. Moreover, they use neural networks to combine both types of data and reverse engineer the best potential origin density landscape (PODLS), which, when used in replication simulations, generates MRT and RFD that are highly correlated with their experimental counterparts. Finally, the authors derive equations to infer intrinsic origin activity (i.e. how often an origin would fire if it were in isolation and therefore immune from inactivation due to passive replication) from observed origin activity. They use discrepancies between intrinsic origin activity and measurements of origin licensing (Orc and Mcm loading) to argue that control at the level of origin firing plays an important role in shaping DNA replication.

Reviewer 3 (Recommendations for the authors):

1) In previously published work with Raji cells (Kirstein et al, 2021), the authors found that ORC and MCM were enriched in early replicating domains and gene promoters and depleted from transcribed genes. However, when controlling for gene transcription and replication timing, they did not observe differences in ORC or MCM abundance between IZ and TZ. Despite the different analytical approaches between their previously published work and the current manuscript, their conclusions that origin licensing is not the sole determinant of replication program are similar.
A) The authors should analyze the same Raji cell datasets using the approach they have developed in the current manuscript.

We have now performed this analysis (Figure 6, Table 1, Table 2, Figure S6, Figures S8, S10, S11, S15, S16).

The PCC between simulated and experimental MRT and RFD data are 0.92 and 0.91, respectively. The MRT PCC is somewhat lower than with other cell lines, probably because the Raji replication timing profile was obtained by sorting cells in 2 compartments of S phase instead of 6 compartments for the other cell lines. The high RFD PCC in contrast reflects the high quality of the Raji OK-seq data and their good consistency with MRT.

The Orc2 and Orc3 data were better predictors of the MRT (PCCs 0.46 -0.50), than the Mcm7 and Mcm3 data (0.19). Therefore the Raji data confirm within a single cell line separate observations made from heterogeneous data (K562 ORC compared with HeLa MCM data). The Raji MRT PCCs are also consistent with the data of Kirstein et al Figure 5a showing that the decrease, from early to late-replicating sequences, in chromatin bound fraction in G1 phase, is stronger for Orc than for Mcm proteins.

The Orc2 and Orc3 data were mediocre albeit significant predictors of the RFD profile (PCCs 0.16) but the Mcm7 and Mcm3 data had no predictive value (0.00). Kirstein et al. concluded that "the densities of ORC/MCM across genomic segments were related to RT but were not predictive of any RFD pattern". Our new results are in agreement for MCM data but apparently detect in ORC data some information that was missed in the cruder, more qualitative analysis of Kirstein et al.

B) Using the analysis in A), a discussion of the relative merits of the two approaches should be provided. Specifically, the approach they have used in the current manuscript is highly intuitive, giving clear expectations and thus allowing drawing clear conclusions.
On the other hand, the previous approach is more difficult to grasp. If the MCM distribution is truly the sole determinant of MRT, why would it be surprising that, after normalizing MCMs for MRT, the difference in MCM abundance at IZ and TZ disappears. (Of note Foss at al 2021 show that IZ exhibit 50% more MCM compared to TZ if no normalization for MRT is carried out).

We thank the reviewer for his/her positive appreciation of the current approach. Strictly speaking, the MCM abundance is only predictive of origin firing time, not of passive replication time. Therefore, one might expect that the replication time of IZs is directly determined by their MCM content, while the replication time of TZs should primarily depend on the MCM content of the nearest IZ. Furthermore, the MCM content of the considered TZ should be significantly lower than that of the nearest IZ, otherwise they would not replicate consecutively.
One concern with the study of Foss et al is that they normalized neither for MRT - although TZs on average replicate later than IZs - nor for transcriptional activity - although IZs are essentially intergenic but often flanked by transcribed genes embedded in adjacent TZs. The significance of the differential MCM content presented by Foss et al is therefore not entirely clear to us.
A general discussion of MCM and ORC abundance profiles is presented lines 644-655 and 710-720.

2) The authors should define RFD, e.g. "RFD is a unitless description of average fork directionality. It varies from -1 to 1, with these extreme values indicating that 100% of the forks are moving leftward

or rightward, respectively.” The authors should also consider adding a supplement in which they briefly describe how RFD is calculated from the OK-seq datasets. They did a terrific job explaining RFD in their Petryk et al. 2016 manuscript, but the reader should not need to read their previous paper to follow the current one.

We have added a brief explanation of OK-seq and a definition of RFD in the Introduction lines 66-72 and in the Materials and Methods lines 820-823 and 846-848.

3) While formulas 1 and 2 are both intuitive and simple to grasp for a biologist with a basic understanding of calculus, this is not the case for formulas 3-8. Before equation 3, the authors should preface things with an explanation of what their analysis to this point has lacked and therefore a motivation for the upcoming derivations of equations 3-8. Then after equation 8, the authors should insert a sentence or two beginning with something like ”The key advantage that we have derived from equations 3 - 8 is that we are now able to ....” This would allow readers who are not interested in the details to skip over the derivations while still grasping the big picture.

The recommended explanations have been added lines 452-460 and 524-526.

4) The authors’ argument that ”... MRT better reflects integrated origin activity over broad regions” hinges on the fact that introducing a gradual up-and-down distortion in the data causes the correlation coefficient between simulated and observed MRT drop more than that for RFD (0.88 to 0.72 versus 0.75 to 0.72, respectively). However, both correlation coefficients end up at the same final level (0.72). It seems illegitimate to make this argument on the basis of the magnitude of the drop in correlation coefficients when they don’t start at the same level. The authors should comment on this.

To address the referee's concern, we have performed similar perturbations of MRT and RFD profiles using DNAse I HSS data as a reference PODLS and the obtained simulated profiles as reference MRT and RFD profiles. When the positions of DNase I HSS were resampled within each 200 kb window, the PCC of the unperturbed and perturbed MRT profiles was 0.98, while the PCC of the unperturbed and perturbed RFD profiles was 0.67. On the other hand, when we modulated the DNase I HSS signal amplitude with a slow varying function of large period (P = 25Mb), the MRT PCC was 0.86 but the RFD PCC was 0.93. Since in this case the starting PCCs are the same (100%), it can be safely concluded that the first perturbation affects RFD much more than MRT, while the second perturbation affects MRT significantly more than RFD.

This novel analysis is now described lines 238-248.

5) The sentence on line 373 should be changed to the following: ”Due to passivation, the observed origin efficiency OE is less than 1.”

The recommended change has been made (line 469).
