# Supplementary material for: Neural network and kinetic modelling of human genome replication reveal replication origin locations and strengths
Source: PLoS Comput Biol. 2023 May 30;19(5):e1011138. doi: 10.1371/journal.pcbi.1011138 (PMC10256156; doi:10.1371/journal.pcbi.1011138)
Supplement: Attachment — Submitted filename: Rebuttal revision Plos OH.pdf [file pcbi.1011138.s003.pdf]

Reviewer's Responses to Questions

Comments to the Authors:Please note here if the review is uploaded as an attachment.

Reviewer #1: The authors have thoroughly and satisfactorily addressed the concerns raised in my previous review.

Reviewer #2: For the previous version of this paper found the reviewers appreciated the usefulness of the approach and the quality of the results but also raised collectively a number of concerns, ranging from technical comments on methods to the reliance on relatively low-quality MCM mapping data in drawing conclusions about the replication program. In the revised version, the authors have done a very good job of responding substantively to the points raised and have improved significantly the paper (likely increasing its impact). The methods of inferring IPLS given here are interesting and fruitful, even if all aspects (such as the form of Eq. (9)) are not fully understood. I suspect that the conclusions as to the variability of MCM firing propensity will continue to be debated, but this paper will be the starting point for any future discussion. I thus recommend publication in PLOS Computational Biology after the following minor points are addressed:

Line 173: “Assuming a linear relation between replication fraction and S-phase duration…”Why? All of the types of models considered have a sigmoidal relation between the S-phase time and replication fraction. Indeed, the discussion in Lines 436-450 concerns just this point….

To check the validity of Eq.1 and Eq.2 we need to have an estimate of the Mean Replication Time in time unit. However experimentaly it is only available in global replicated fraction. This is why we need this assumption. To account for the reviewer comment we modified the sentence into:

Line 173 : To experimentally check Equation (1) we need to convert Repli-seq data, expressed in replicated genome fraction, into time units by multiplication by an estimate of S-phase duration TS. This implicitly assumes a linear relation between replicated fraction and time in S phase, although the true relation appears to be sigmoid rather than linear, which may slightly distort very early or very late S-phase data.

Line 178: “… circumvented by data smoothing…”This is precisely what Eq. (2) does (at a length scale l)

And this is what we had in mind. We clarified it as follows:

Line 181 : This can be circumvented by data smoothing at the expense of resolution by integrating Eq. (1) at point x over length l leading to:

Line 251: “deriving RFD profile from MRT data Eq. (1) by numerical derivative would produce low resolution RFD profiles with amplified noise….”— again, not true if the derivatives were estimated in a more sophisticated way.

Here the low resolution of the RFD profiles obtained here is mainly due to the low resolution of MRT data. We clarified this as follows:

Line 253 : Deriving RFD profiles from limited resolution (~100 kb) MRT data would produce low resolution RFD profiles

For Lines 178, 251, there is nothing wrong with methods used and I agree that MRT is better at long and RFD at short scales, but it’s also true that there is no real advantage of using Eq. (2) rather than Eq. (1) with derivatives smooth over the same length scale.

We agree with this comment, but think that using Eq 2 makes clearer the smoothing process for people without knowledge on the different methods to estimate the derivatives

Line 505: Taylor expansion (not extension)

We have corrected this.

Lines 517-520 and Eq. (9): The authors’ arguments are superficially reasonable, and I am willing to let the point stand. However, the good agreement between the ad hoc exponential in Eq. (9) seen in Fig. 8C, supposedly from combining the time-variation of Ffree(t) with the expected 1/t dependence, makes one wonder….

Unfortunately we were not able to theoretically determine the origin of this agreement.

• It might be clearer to write I_M(x) rather than I_M to emphasize that the profile is something that varies along the genome, as opposed to other quantities such as fork velocity that are assumed constant over the genome. (The x-dependence is occasionally given, as in Line 481, but not usually.)

We changed I_M to I_M(x) or I_M profile in most occurrences of I_M to highlight the spatial dependency

• The phrase “reciprocally consistent” should probably be “mutually consistent” as the former suggests MRT and RFD might be inversely related, which is not what is intended.

We changed to mutually consistent. (line 670)

Reviewer #3: In the revised manuscript the authors have addressed most of the concerns that were raised in the initial review. The overall performance of their models is impressive, and their results provide a solid foundation on which other researchers can build.However, I suggest that the authors:1) discuss the inconsistencies of the measurements of ORC and MCM components and the difficulties that these inconsistencies create when attempting to draw conclusions about the relative contributions of origin licensing vs firing and2) clarify the role of “confounding parameters” such as transcription and MRT when discounting origin density model.

1. One of the goals of the study is to help elucidate the relative contribution of origin density vs. origin affinity in shaping genome replication in human cells. While the authors' replication modeling performs well at both large (MRT) and smaller scales (RFD), the final determination of the origin affinity vs origin density critically depends on having accurate measurements of ORC and MCM density across the genome. In Table 1, correlations for ORC are as high as 0.87 (for MRT) and 0.74 (for RFD) in K562 cells and lower but still positive at 0.46 and 0.16 in Raji cells. For members of the MCM complex such correlations were all over the place: from relatively high positive correlations: 0.52 (MRT) and 0.41 (RFD) for MCM2 in HeLa cells; no correlations 0.19 (MRT) and 0.00 (RFD) for MCM3 in Raji; to high negative correlations -0.80 (MRT) and -0.22 (MRT) for hMCM-DH. Which one of these MCM measurements is true? The authors recognize robust correlations of ORC density with both MRT and RFD based on high positive correlations for ORC2 in HeLa cells but dismiss the correlation between MCM and MRT and RDF even though the correlations for MCM2 are higher at 0.52 (MRT) and 0.41(RFD) in HeLa cells compared to ORC2 correlations of 0.46(MRT) and 0.16(RFD) in Raji cells. Given inconsistencies in MCM measurements, it is difficult to draw firm conclusions about licensing vs firing models based on these results and the authors should discuss these limitations.

In the discussion we added :

Line 660 : However, the high variability of MCM profiles prevents us to totally exclude that when the source of this variability is understood, an improved MCM profile may better predict RFD and MRT.

Lines 123 to 125 The authors write: “However, our comparison of ORC, MCM and RFD profiles of the Raji cell line showed that when confounding parameters such as MRT and transcription status are controlled, ORC and MCM densities are not predictive of Izs.”

Based on the absence of correlations of MCM3 and RFD in Raji cells R=0.00, it is not surprising that MCM3 does not delineate IZs in their previous study (Kirstein et al 2021), whether or not confounding parameters are taken into account. On the other hand, replication correlated with MCM density based on MCM2 density measurements (Foss 2021) for both MRT (0.52) and RFD(0.41), which could perhaps explain higher MCM density in IZs in that study.Finally, why would one need to take transcription status into account in determining origin density vs origin affinity model. If MCM densities were the sole determinant of replication initiation, and the MCM densities are a reflection of transcription (i.e. MCM are not found within transcribed genes), removing transcription as a “confounding parameter” would immediately discount the origin density model.

We agree with these remarks. To avoid any confusion we have rephrased the sentence as:

line 123 : However, our comparison of ORC, MCM and RFD profiles of the Raji cell line showed that at constant MRT and transcription level, ORC and MCM densities are similar in initiation, random replication and termination zones.

Have the authors made all data and (if applicable) computational code underlying the findings in their manuscript fully available?The PLOS Data policy requires authors to make all data and code underlying the findings described in their manuscript fully available without restriction, with rare exception (please refer to the Data Availability Statement in the manuscript PDF file). The data and code should be provided as part of the manuscript or its supporting information, or deposited to a public repository. For example, in addition to summary statistics, the data points behind means, medians and variance measures should be available. If there are restrictions on publicly sharing data or code —e.g. participant privacy or use of data from a third party—those must be specified.

Reviewer #1: Yes

Reviewer #2: Yes

Reviewer #3: Yes

PLOS authors have the option to publish the peer review history of their article (what does this mean?). If published, this will include your full peer review and any attached files.If you choose “no”, your identity will remain anonymous but your review may still be made public.**Do you want your identity to be public for this peer review?** For information about this choice, including consent withdrawal, please see our Privacy Policy.

Reviewer #1: No

Reviewer #2: No

Reviewer #3: No
